# Legumes Can Increase Cadmium Contamination in Neighboring Crops

**DOI:** 10.1371/journal.pone.0042944

**Published:** 2012-08-14

**Authors:** Ling Liu, Qian Zhang, Liangliang Hu, Jianjun Tang, Ligen Xu, Xiantian Yang, Jean W. H. Yong, Xin Chen

**Affiliations:** 1 College of Life Science, Zhejiang University, Hangzhou, China; 2 Singapore University of Technology and Design, Singapore, Singapore; 3 Taizhou Academy of Agriculture Sciences, Taizhou, China; Kansas State University, United States of America

## Abstract

Legumes are widely used in many cropping systems because they share their nitrogen fixation products and phosphorus mobilization activities with their neighbors. In the current study, however, we showed that co-cultivation with legumes increased cadmium (Cd) contamination in the adjacent crops. Both field and mesocosm experiments indicated that legumes increased Cd levels in edible parts and shoots of four neighboring crops and five maize varieties tested, regardless of the Cd levels in the soil. This enhanced Cd accumulation in crops was attributed to root interactions that alter the rhizosphere environment. Co-cultivation with legumes reduced soil pH, which somewhat increased the exchangeable forms of Cd. Our results have demonstrated the inevitable increases in Cd levels of crops as a direct result of co-cultivation with legumes even under situations when these levels are below the permissible threshold. With this new revelation, we need to consider carefully the current cropping systems involving legumes and perhaps to re-design the current and future cropping systems in view of avoiding food contamination by Cd.

## Introduction

Legumes are good partner plants in natural plant communities because legumes can accumulate soil N and improve soil conditions for their neighbors [1]. Legume plants are widely used as nurse plants for degraded land restoration [2]–[4]. In agriculture, because legumes fix nitrogen and solubilize phosphorus, therefore they are often used to improve the growth of partner crops and are widely used in many intercropping systems and crop rotation cycles, especially in poor soils [5]–[7]. Legume-based cropping systems are considered the better way to increase productivity of the land and to maintain soil fertility [5]. In China, more than ten million hectares of legume-based intercropping or crop rotation are planted each year [7]. Legume-based cropping systems are also becoming common in other parts of the world such as India, Southeast Asia, South America, Central America, and Africa [8]. Legume-based cropping systems are also widely practiced in North America [9] and the European countries [10].

However, recent studies showed that legumes can also enhance Cd uptake of their neighboring plants. When co-planted with legumes maize can accumulate more Cd in its leaf, stem and root [11]. Legume can increase Cd concentration and total Cd accumulation of the co-cultivated tobacco [12]. Cadmium concentration in the grains was also highest in the wheat crop which was grown immediately after a crop rotation cycle with lupins as the predecessor [13]. As soil pollution by heavy metals is an inevitable problem associated with the economic and agricultural development in many countries, there are concerns that legumes enhance the risk of their neighboring crops growing in soils contaminated with varying levels of cadmium in any form of legume-based intercropping systems.

Therefore, we conducted field and greenhouse experiments to examine whether legumes increase Cd accumulation in the edible parts of their neighboring crops. We first compared Cd concentration in edible parts of four crops grown in monoculture and in mixture with three legumes through field experiments. Following which, we conducted a mesocosm experiment to further examine whether legume can increase Cd concentration in the edible parts by using three Cd levels in soil and five different maize varieties. In the microcosm experiment, we examined the changes in pH and concentration of exchangeable Cd fractions in rhizosphere when the crops are co-planted with legumes.

## Results

### Field study

**Figure 1:**
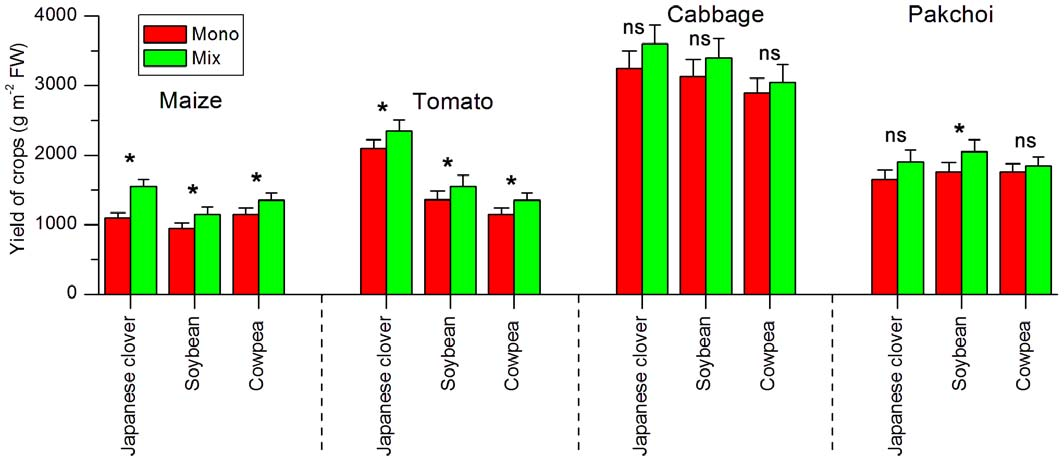
Yield of the edible parts of four crops grown in monoculture and mixture with Japanese clover, soybean and cowpea. Values are means (± SE) of four replicate plots. For each crop, values with asterisk (*) are significantly different (LSD at P<0.05).

In field experiments, yields of maize and tomato increased (P<0.05) but yields of cabbage and pakchoi did not changed (P>0.05) under mixed planting with legumes when compared to monocultures (Fig. 1).

**Figure 2:**
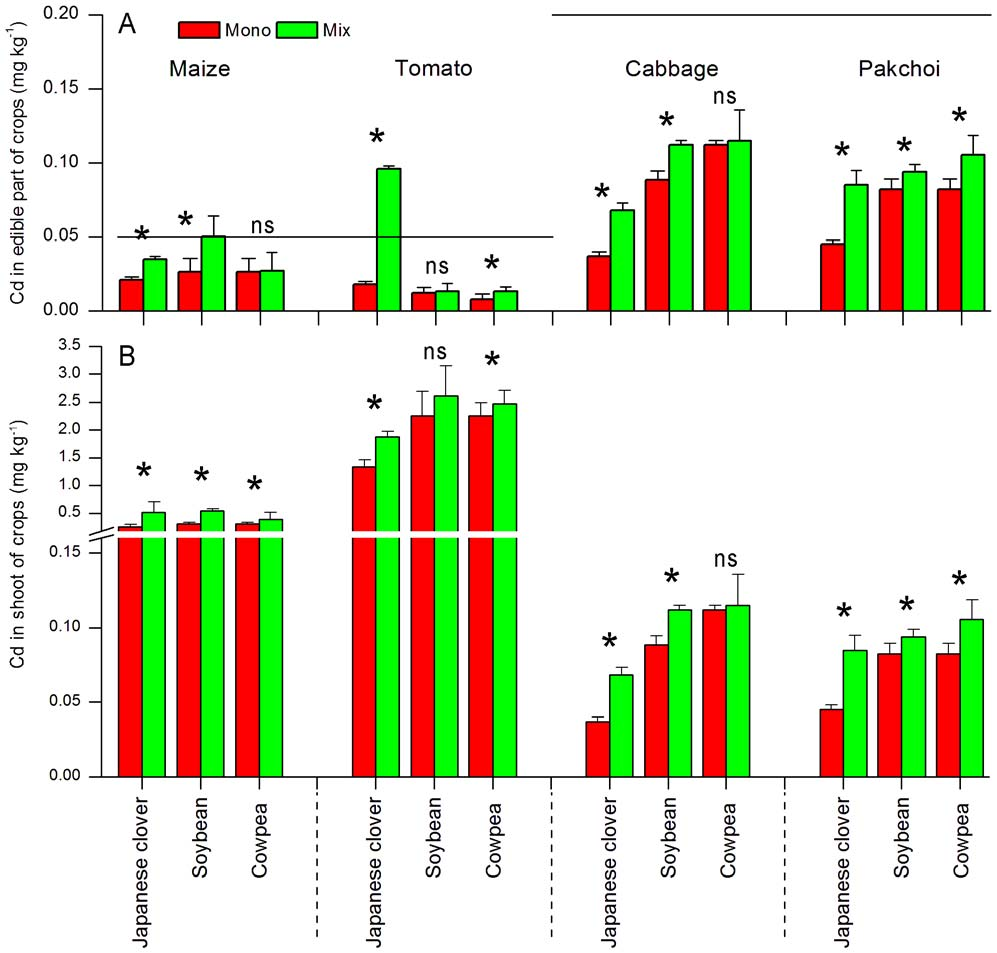
Cd concentrations in the edible parts (A) and shoots (B) of four crops grown in monoculture and mixture with Japanese clover, soybean and cowpea. The lower and upper horizontal lines indicate the Cd MPC for fruit-type and leafy vegetables, respectively. Values are means (± SE) of four replicate plots. For each crop, values with asterisk (*) are significantly different (LSD at P<0.05).

When using Japanese clover as a partner crop, planting pattern caused significant changes (P<0.01) in Cd concentration of edible parts for the four crops (maize, tomato, cabbage and pakchoi). Specifically, the levels of Cd were significantly higher when crop plants were planted with Japanese clover than when the crops were grown alone (Fig. 2A). For tomato, Cd concentration of edible part was below the maximum permissible concentration (MPC) standard of the National Standard Agency in China [14] when grown alone, but above the MPC when co-planted with legume (Fig. 2A). Using soybean as a partner crop, the mixed planting approach increased Cd concentration of the edible parts of maize, cabbage and pakchoi (P<0.05) with the exception for tomato (P>0.05), when compared to the plants in monoculture (Fig. 2A). When the main crops were grown with cowpea, the Cd concentration of edible parts of tomato and pakchoi increased (P<0.05), whereas Cd levels of edible parts of maize and cabbage remained at similar levels (P>0.05) (Fig. 2A).

Japanese clover, soybean and cowpea also significantly increased Cd accumulation in the shoots of maize (P<0.05, Fig. 2B). For tomato, soybean did not significantly increase (P>0.05), but Japanese clover and cowpea significantly increased (P<0.05) Cd accumulation in shoot (Fig. 2B). Effects of the three legumes on Cd accumulation in shoots of cabbage and pakchoi were similar to the effects in edible parts. (Fig. 2B).

There were no significant differences for the total and exchangeable Cd in soils between monoculture and intercropping after each experiment (Table S1).

### Mesocosm study

**Figure 3:**
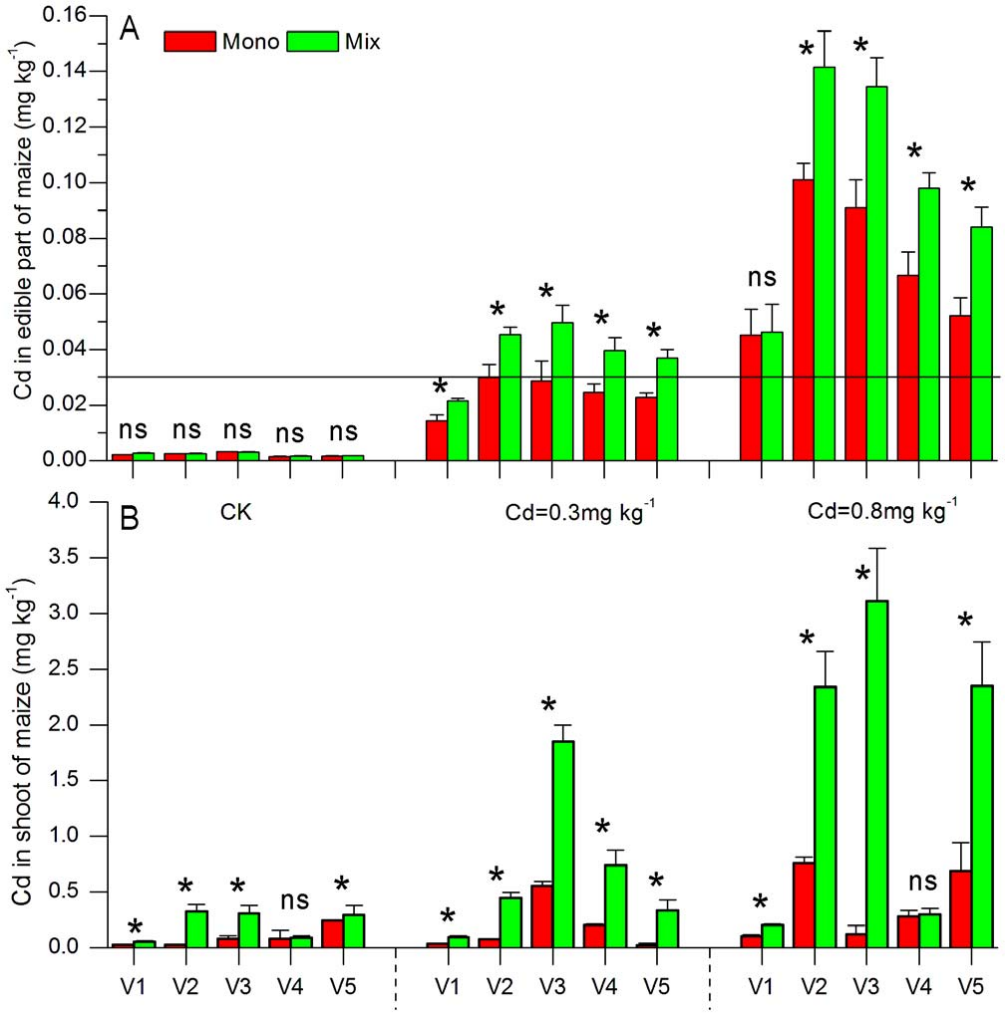
Cd concentrations in edible parts (A) and shoots (B) of five maize genotypes grown under monoculture and mixture. V1 (Zhengdan-958), V2 (Suyunuo-2), V3 (Huyunuo-2), V4 (Jinzhumichaotian), V5 (Shentian-1) indicate the different maize genotypes. The horizontal line indicates the Cd MPC for maize. Values are means (±SE) of four replications. Values with asterisk (*) are significantly different (LSD at P<0.05).

In mesocosm experiment, soil Cd concentrations significantly impacted Cd concentration of edible part of the five maize varieties (for variety Zhengdan-958, F_2,20_ = 68.130, P = 0.000; for variety Suyunuo-2, F_2,20_ = 197.763, P = 0.000; for variety Huyunuo-2, F_2,20_ = 135.844, P = 0.000; for variety Jinzhumichaotian, F_2,20_ = 96.257, P = 0.000; for variety Shentian-1, F_2,20_ = 5.538, P = 0.012). Cd concentrations in edible parts increased with the soil Cd concentrations (Fig. 3A). While the planting patterns did not change Cd concentration in edible parts of variety Zhengdan-958 (F_1,20_ = 0.810, P = 0.379), planting patterns significantly altered the Cd concentration in edible parts of the other four varieties (for variety Suyunuo-2, F_1,20_ = 13.675, P = 0.001; for variety Huyunuo-2, F_1,20_ = 14.649, P = 0.001; for variety Jinzhumichaotian, F_1,20_ = 18.271, P = 0.000; for variety Shentian-1, F_1,20_ = 5.179, P = 0.027). Cd concentrations in edible parts of the four varieties were significantly higher under mixed planting with legume than that when growing under monoculture for all Cd treatments (P<0.05) (Fig. 3A).

Except for the variety Jinzhumichaotian (V4) at control and 0.8 mg kg^−1^ Cd treatment, Cd accumulation in shoots of the other maize varieties was significantly enhanced when co-planted with Japanese clover across the three soil Cd levels (P<0.05, (Fig. 3B).

### Microcosm study

**Figure 4:**
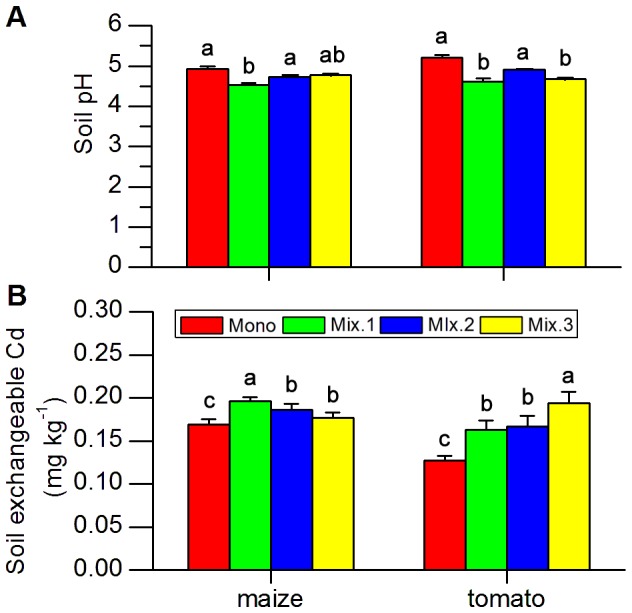
Soil pH (A) and Cd concentrations of exchangeable form (B) in soil grown under monoculture and intercropping. Mono.: crop monoculture. Mix.1: co-planted with Japanese clover. Mix. 2: co-planted with soybean. Mix.3: co-planted with cowpea. Values are means (±SE) of four replications. Values with different letters are significantly different (LSD at P<0.05).

Soil pH of both crops (maize and tomato) was significantly influenced by planting patterns (for maize, F_3,12_ = 11.231, P = 0.001; for tomato F_3,12_ = 36.166, P = 0.000). Compared to monoculture, Soil pH significantly decreased when either maize or tomato were planted with Japanese clover and cowpea (Fig. 4A, P<0.05), but soil pH did not change when maize or tomato plants were co-planted with soybean (Fig. 4A, P>0.05).

Planting patterns also significantly impacted the levels of Cd exchangeable fractions in soil for both maize and tomato planting system (for maize, F_3,12_ = 10.507, P = 0.001; for tomato F_3,12_ = 23.700, P = 0.001). For maize, plants co-planted with Japanese clover and soybean (P<0.05) significantly increased the Cd exchangeable fraction in soil (Fig. 4B, P<0.05) but not with cowpea (Fig. 4B, P>0.05). All the three legumes increased the Cd levels of exchangeable fraction in rhizosphere soil when co-planted with tomato (Fig. 4B, P<0.05).

## Discussion

In our field experiment when Cd concentration (0.283 mg kg^−1^) in soil was under the permissible limiting concentration [15], the yields of all crops using the legume-crop co-planting approach generally increased when compared to monoculture. However, Cd levels of edible parts in all four crops (maize, tomato, cabbage and pakchoi) were higher when the crops were co-cultivated with legumes than when planted alone. The edible parts of tomato grown in monoculture had Cd levels which were below the maximum permissible concentration (MPC) standard of the National Standard in China [14], but these levels exceeded the MPC [14] when co-cultivated with legumes. Our mesocosm experiment further ascertained the fact that legume increased Cd accumulation in the edible parts of its neighbors. For the mesocosm experiment, co-planting with legumes increased Cd concentrations in edible parts of all five maize varieties regardless of the Cd levels (non-polluted, 0.047 mg kg^−1^ Cd; slightly polluted, 0.3 mg kg^−1^ Cd; and moderately polluted, 0.8 mg kg^−1^ Cd) in the soil. These results suggested that legumes may enhance the risk of crop foods contaminated by Cd even though the Cd concentration in the soil is under the permissible limiting concentration.

There are two plausible mechanisms that could explain why legumes can enhance Cd accumulation in the edible parts of the neighbor crops. Firstly, legumes may increase the total Cd uptake of neighboring crops through root interaction. Secondly, legumes may alter Cd allocation to different parts of the crops [12] (Liu et al., 2011). In our field experiments, Cd accumulation in shoots of crops was significantly higher when co-planted with legumes compared to crops grown alone. Our mesocosm study on the five genotypes of maize also showed that legumes significantly increased Cd accumulation in shoots. These results suggested that the enhancement of Cd uptake by legumes played a major role in enhancing Cd accumulation in the edible parts of the crops.

**Figure 5:**
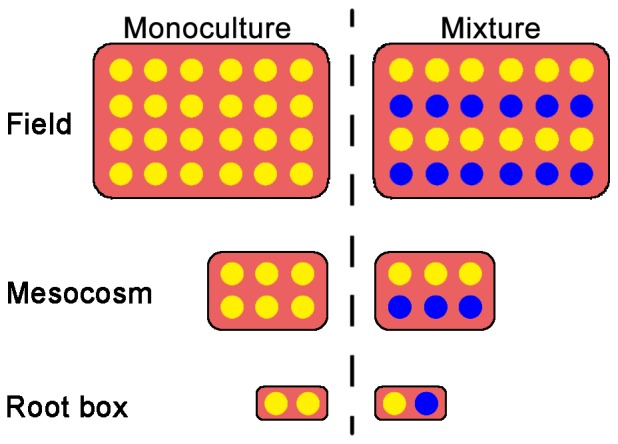
Experimental design. Yellow dot refers to the test crop plant; blue dot refers to the interplanted legume plant. For maize and tomato, row space and distance between plants within row were 45 cm and 30 cm respectively; for cabbage and pakchoi, row space and distance between plants within row were 35 cm and 20 cm respectively.

Results from our microcosm study further suggested that root interactions between the co-existing crops could cause the significant increases in Cd accumulation. Plants can only absorb Cd in the exchangeable form, which usually constitutes a small portion of the total Cd content in soils [16]. However in the rhizosphere, there are two factors that could increase the exchangeable form of Cd. One is the presence of low molecular weight organic acids (e.g., acetic, oxalic, fumaric, citric, and tartaric acids) which form soluble complexes with Cd, thus releasing Cd from the soil matrix [17]. The other is attributed to the low soil pH values which desorbs the Cd from absorbed forms in soils [18], [19]. Legumes are well known to reduce pH values because these plants exude large quantities of H^+^ during the process of nitrogen fixation. Consequently, localized soil acidification occurs when crops are co-planted with legumes [5]. When maize or tomato was inter-planted with legumes (Japanese clover and cowpea) in our microcosm experiment, soil pH significantly decreased (Fig. 4) with a concomitantly increased in soil exchangeable Cd levels (Fig. 5). In our microcosm study, soybean did not reduce soil pH, but still increased the exchangeable Cd in rhizosphere soil of the soybean-crop system. At present, it remains unclear whether low molecular weight organic acids play a role in determining Cd exchangeability in the soil, and more research is needed to clarify this phenomenon.

Our study indicated that the enhanced Cd accumulation in neighbor crops (both the edible part and shoot) by legumes varied in specific legume-crop system. Cd accumulation in all partner crops used in the study was increased by Japanese clover. This could be attributed to the fact that Japanese clover can acidify rhizosphere soil and leading to higher levels of exchangeable Cd [12] (Fig.4 and Fig. 5). Although soybean did not reduce soil pH, it was able to enhance Cd accumulation in three crops but not for tomato. Contrary to soybean, cowpea reduced the pH in soils of all crops tested and affected Cd accumulation in tomato and pakchoi. Thus, the exact mechanism(s) governing Cd accumulation for each legume-crop pair needs further research.

Soil polluted by heavy metals and metalloids is an increasing problem in many developing countries, resulting from increased industrial activities and chemical fertilizer utilization [20]. In these countries, the increasing use of synthetic nitrogen fertilizers to drive higher crop yield has also resulted in soil N depletion [21]. Alternative approaches to producing more food while maintaining soil fertility are urgently needed in view of decreasing farmlands [22]–[26]. Legume-based intercropping has been widely promoted as one of the better alternate strategy to increase crop production and maintaining soil fertility [5], [22]. However, our results do not support growing legumes in soils with elevated Cd levels even though these Cd levels are near to or below the pollution threshold as the co-cultivated legumes will biologically increase the Cd level in the adjacent crop. This new revelation suggests that the future design of cropping systems involving legumes should be carefully considered to avoid food contamination by Cd although legumes and crops vary in responding to a specific legume -crop system.

## Materials and Methods

### Field Study

#### Ethics statement

The field study was conducted in the area that corn and vegetable are main crops. It locates in Taizhou City (28°30′N, 121°22′E), Zhejiang Province, China. The field for the experiments is belong to the Long-term Experimental Station of Taizhou Academy of Agriculture Sciences, which is authorized by the government of Taizhou City, Zhejiang Province, China. The field is not privately-owned or protected. No specific permits were required for the described field studies. During the experiment, no other specific permission was required because we only carried out normal agricultural activities. Also our field study did not involve endangered or protected species.

#### Climate and soil

The climate in the field study area is typically of the subtropical monsoon with a mean annual air temperature of 18.6°C and a mean annual precipitation of 1693 mm. The soil was a sandy loam with a pH of 5.6. The soil contained 38.1 g kg^−1^ organic matter, 2.01 g kg^−1^ total nitrogen (N), 183.3 mg kg^−1^ extractable phosphorus (P), and 106.8 mg kg^−1^ extractable potassium (K) (extracted by 2 M HNO_3_ and determined by flame atomic absorption spectrophotometry, flame-AAS). Total and exchangeable Cd concentrations in the soil was 0.283 and 0.128 mg kg^−1^ respectively (determined by Inductively Coupled Plasma Mass Spectrometry, ICP-MS), which is considered to be slightly polluted but is below the limit for Cd as indicated by the Environmental Quality Standards set by the State Environmental Protection Administration for soils in China [15].

#### Legumes and crops

Four crops and three legumes that are cultivated extensively in the local region were used in the field study. The four crop species were maize (*Zea mays* L. var. Shentian-1), tomato (*Solanum lycopersicum* L. var. Zhongshu-4), cabbage (*Brassica oleracea* L. var. Jingfeng-1), and pakchoi (*Brassica chinensis* (L.) Makino. var. Youdonger-Hangzhou). The three legumes used as partner plants were Japanese clover [*Kummerowia striata* (Thunb.) Schindl.], soybean (*Glycine max*(L.)Merr.) and cowpea (*Vigna unguiculata* (Linn.) Walp.). Japanese clover is a common legume cover crop that is planted as forage, and soybean and cowpea are common legume crops in this area.

#### Experimental design

Three experiments were designed to test the effects of the three partner legumes on their neighboring crops at the same field (Fig. 5) in different years. In each experiment, there were nine treatments (one legume monoculture and four crop monoculture, and four interplanting). Each of the nine treatments was represented by four replicate plots (3×5 m per plot), and the 36 plots were arranged in randomized complete blocks.

The three experiments were conducted separately by using Japanese clover, or soybean, or cowpea as one of the intercrops in 2009, 2010 and 2011 respectively. In early March, the seeds of tomato, cabbage, pakchoi and legume partner were germinated and pre-cultured in the plastic mesh plate with vermiculite and peat in greenhouse under natural light and temperature. After 40 days, the pre-cultured seedlings were transplanted and the seeds of maize were sown simultaneously. Solid compound fertilizer (N:P:K = 15∶15∶15) was applied at 250 kg ha^−1^ in all plots for the duration of the experiment.

#### Measurements

Pakchoi was harvested 70 days after planting, and tomato and cabbage were harvested 95 days after transplanting, and maize was harvested 105 days after sowing. Edible parts of tomato and maize were harvested at their mature stage. Ten plants were randomly sampled in each plot for determination of crop yield and for quantification of Cd in shoots and the edible part. For pakchoi and cabbage, both the edible part and shoot are the leaves; for maize, the edible part is the ear without husk; for tomato, the edible part is the fresh fruit.

The edible parts of the crops were briefly washed with tap water and then with deionized water to remove surface dust and soil. After the fresh weights were recorded, the tissues (except the edible part of tomato) were dried at 80°C for 48 h and weighed. The fresh tomato fruit were processed in a blender and kept in clean polyethylene bags for metal analysis. Samples of edible crop parts that were used for the determination of Cd concentration were dried and ground to <0.25 mm in a stainless steel mill. Subsamples (1.0 g) of plant tissues were digested with concentrated HNO_3_ and HClO_4_ at a ratio of 4∶1 (*V/V*), and the digest was adjusted to a final volume of 50 mL with deionized water. The concentrations of metals in the solutions extracted from the plant tissues were analyzed by ICP-MS (PerkinElmer, ELAN DRC-e, USA). The accuracy and reproducibility of the method for Cd in crop edible parts were tested by using a plant standard reference material Rice Powder GBW08502 [27]. The Cd content in the edible parts of crops was calculated on a per unit fresh weight basis by using the ratio of dry weight and fresh weight.

After each experiment, soil samples were collected from five locations in each plot. Total and exchangeable Cd concentrations in the soils were determined by Inductively Coupled Plasma Mass Spectrometry (ICP-MS).

#### Statistic analysis

In each legume experiment, one-way ANOVA was performed to determine the effect of planting patterns (monoculture or mixture with legume) on Cd concentration in the shoot and edible part for each crop, and on total and extractable Cd concentrations in soils after each experiment. The general linear model (GLM) in the SPSS V.10.0 (SPSS Inc., Chicago, USA) was used, and means were compared with the least significant difference at the 5% confidence level.

### Mesocosm study

Mesoscosm experiments were conducted to verify that the effects of the legumes on neighboring crops to accumulate Cd were not by chance. The experimental soil was collected from the surface layer (0–20 cm) of an unpolluted vegetable field in Hangzhou City (28°54′N, 111°30′E), Zhejiang Province, in southeastern China. The basic properties of the soil were pH 5.9, organic matter content 38.6 g kg^−1^, total N 2.49 g kg^−1^, extractable P 98.03 mg kg^−1^, extractable K 264.72 mg kg^−1^, and total Cd 0.047 mg kg^−1^. There were four replicates with five maize varieties, grown under two planting patterns (monoculture or mixture, Fig. 5). Japanese clover was used as partner legume. The five varieties were Zhengdan-958 (V1), Suyunuo-2 (V2), Huyunuo-2 (V3), Jinzhumichaotian (V4), and Shentian-1 (V5). Cadmium was added in the form of 3CdSO_4_·8H_2_O and Cd treatments of 0.0, 0.3, or 0.8 mg Cd per kg of soil were used to simulate soil without Cd contamination, with slight Cd contamination, and moderate Cd contamination, respectively. The mesocosms were plastic containers that were 42 cm long, 32 cm wide and 20 cm deep. Each mesocosm was filled with 20 kg of well-mixed soil. Soil moisture content was adjusted to about 60% of water-holding capacity based on weight and the soil was placed in the mesocosm one month before planting to allow various sorption mechanisms and other processes to stabilize. During that month, water was added to maintain soil moisture at 60% of water-holding capacity. Mesocosms were arranged in a greenhouse in a randomized complete block design. Plants were maintained under natural light and temperature conditions with an average air temperature of 18–30°C during the course of the experiment from April to August. Plants were watered daily to maintain soil moisture at 70–90% of water-holding capacity. No additional nutrients were applied.

The edible part of each maize variety was harvested at the mature stage, and Cd concentrations were determined as described for the field experiment.

For each maize variety, two-way ANOVA was performed to determine the effects of planting patterns and soil Cd level on Cd concentration in the edible part. The general linear model (GLM) in the SPSS V.10.0 (SPSS Inc., Chicago, USA) was used, and means were compared with the least significant difference at the 5% confidence level.

### Microcosm Study

To test the hypothesis that legumes enhanced Cd accumulation in their neighbors by changing the rhizosphere environment, we conducted a microcosm experiment by using the same three legumes mentioned in the field experiment. The two crops used were maize var. Shentian-1 and tomato var. Zhongshu-4, grown with three partner legumes (Japanese clover, soybean and cowpea) under two plant patterns (monoculture and mixture) (Fig. 5).

The soil used for the microcosm experiment was collected from the surface layer (0–20 cm) of field experiment location. The soil was collected, air dried, mixed, crushed, and passed through a 5-mm sieve. The basic properties and Cd contents of the soil were the same as the field experiment. Plastic root boxes of 22 cm length, 14 cm width, and 15 cm depth used in the microcosm experiment. Each root box was filled with 4 kg of well-mixed soil. Root boxes were arranged in the greenhouse in a complete randomized block design with four replicates. Plants were maintained under the conditions as described by the mesocosm experiment. No additional nutrients were applied.

Soil samples of 50 g were collected 15 days after the first flowering of the maize and tomato using a soil sampling tube from five locations in each microcosm. Soil pH was measured in a 1∶5 (*W/V*) suspension of soil and deionized water. The chemical forms of Cd in soil were quantified by a sequential extraction procedure [27] and were analyzed by flame-AAS.

For each crop (maize or tomato), a two-way ANOVA was performed to determine the effects of legumes, planting patterns on soil pH and exchangeable forms of Cd in each rhizosphere soil of crops. The general linear model (GLM) in the SPSS V.10.0 (SPSS Inc., Chicago, USA) was used, and means were compared with the least significant difference at the 5% confidence level.

## Supporting Information

Table S1 Total and exchangeable Cd in soils after each experiment.
(DOC)
