# Supplementary material for: Legumes Can Increase Cadmium Contamination in Neighboring Crops
Source: PLoS One. 2012 Aug 14;7(8):e42944. doi: 10.1371/journal.pone.0042944 (PMC3419222; doi:10.1371/journal.pone.0042944)
Supplement: Table S1 — Total and exchangeable Cd in soils after each experiment. (DOC) [file pone.0042944.s001.doc]

**Supporting information**

**Table S1**

|  | Total Cd in soil  (mg kg^-1^) | | Exchangeable Cd in soil  (mg kg^-1^) | |
| --- | --- | --- | --- | --- |
|  | Monoculture | Intercropping | Monoculture | Intercropping |
| Japanese clover  Maize  Tomato  Cabbage  Pakchoi | 0.253±0.048a  0.241±0.015a  0.269±0.001a  0.233±0.080a | 0.251±0.019a  0.245±0.020a  0.267±0.008a  0.228±0.016a | 0.115±0.014a  0.124±0.009a  0.133±0.010a  0.118±0.009a | 0.123±0.014a  0.128±0.011a  0.135±0.014a  0.115±0.013a |
| Soybean  Maize  Tomato  Cabbage  Pakchoi | 0.233±0.021a  0.232±0.012a  0.236±0.006a  0.228±0.016a | 0.223±0.010a  0.225±0.018a  0.210±0.003a  0.218±0.039a | 0.131±0.019a  0.120±0.020a  0.110±0.008a  0.120±0.016a | 0.123±0.010a  0.125±0.011a  0.113±0.001a  0.123±0.012a |
| Cowpea  Maize  Tomato  Cabbage  Pakchoi | 0.236±0.019a  0.192±0.020a  0.238±0.008a  0.228±0.016a | 0.238±0.020a  0.189±0.002a  0.230±0.001a  0.223±0.003a | 0.123±0.010a  0.099±0.012a  0.136±0.010a  0.123±0.012a | 0.120±0.010a  0.094±0.013a  0.131±0.012a  0.125±0.011a |
